# Factors associated with lower extremity atherosclerotic disease in Chinese patients with type 2 diabetes mellitus: A case-control study

**DOI:** 10.1097/MD.0000000000005230

**Published:** 2016-12-23

**Authors:** Qingge Gao, Binbin He, Chaoyu Zhu, Yuanyuan Xiao, Li Wei, Weiping Jia

**Affiliations:** Department of Endocrinology and Metabolism, Shanghai No. 6 People's Hospital Affiliated to Jiaotong University, Shanghai, China.

**Keywords:** glomerular filtration rate, lower extremity atherosclerotic disease, risk factors, type 2 diabetes mellitus, uric acid

## Abstract

Early detection and treatment of lower extremity atherosclerotic disease (LEAD), and controlling its risk factors are critical in preventing amputation and death in diabetic patients. This study aimed to investigate the factors associated with LEAD in Chinese diabetic patients.

In this case-control study, patients with type 2 diabetes mellitus (T2DM) (N = 1289) were divided into 2 groups according to the ultrasonic Doppler examination: with (LEAD+, n = 737) and without (LEAD−, n = 552) LEAD. In subgroup analysis, the LEAD+ group was divided based on the diameter of lower-extremity arteries: LEAD+A (1%–49% reduction) and LEAD+B (≥50% reduction). Clinical and demographic data of patients were analyzed.

Compared with the LEAD− group, serum creatinine levels were significantly increased (*P* < 0.001), whereas glomerular filtration rate (GFR) was significantly decreased (*P* < 0.001) in the LEAD+ group. Multivariate analysis results showed that GFR (odds ratio [OR] 0.991, 95% confidence interval [CI] 0.986–0.997, *P* = 0.003), diabetes duration (OR 1.055, 95% CI 1.026–1.084, *P* < 0.001), age (OR 1.123, 95% CI 1.104–1.142, *P* < 0.001), and uric acid (OR 1.002, 95% CI 1.000–1.004, *P* = 0.031) were independently associated with LEAD in patients with T2DM. Furthermore, multivariate analysis showed that age (OR 1.078, 95% CI 1.048–1.109, *P* < 0.001) and GFR (OR 0.985, 95% CI 0.975–0.994, *P* = 0.002) were independently associated with the severity of arterial lesions in patients with T2DM and LEAD.

The risk factors of LEAD in Chinese patients with T2DM include age, course of disease, uric acid, and GFR. Patients with T2DM, high uric acid levels, and declined GFR could be listed in the high-risk group for LEAD.

## 1 Introduction

Type 2 diabetes mellitus (T2DM) incidence is increasing in China at an alarming rate, imposing a considerable burden on public health. The latest epidemiological survey showed that the diabetes prevalence in individuals above 20 years old was 9.7%, with a prediabetes prevalence reaching 15.5% in the Chinese population.^[1]^ The common acute complications of T2DM include diabetic ketoacidosis, hyperglycemia, hypoglycemia, and diabetic coma. The major chronic complications include microangiopathies (ie, diabetic cardiomyopathy, neuropathy, nephropathy, and retinopathy), macrovascular diseases (ie, coronary artery and peripheral vascular diseases, and stroke), diabetic foot, necrosis, and gangrene. Atherosclerosis is a progressive disease and its clinical consequences are potentially life-threatening.

As a frequently encountered macrovascular complication of diabetes, lower extremity atherosclerotic disease (LEAD) counts among the main factors causing foot ulceration and amputation,^[2]^ which lead to markedly reduced quality of life.^[3]^ Moreover, LEAD constitutes an important indicator of atherosclerotic disease burden, and is associated with high mortality from cardiovascular and cerebrovascular causes.^[4]^ Peripheral artery disease is characterized by occlusion of lower-extremity arteries,^[5]^ which results in functional impairment,^[6]^ disability,^[7]^ foot ulceration, and lower-extremity amputation.^[8]^ Diabetic patients are 15 times more likely to suffer from lower-extremity amputation than people without diabetes.^[9]^ Epidemiological data indicated that the incidence of diabetic LEAD in the Asian population is different from that obtained for other populations around the world. Umuerri and Obasohan^[10]^ reported a LEAD prevalence of 35.6% in Nigeria. Another study assessing patients in Costa Rica found an incidence rate for diabetic LEAD of 6.02 per 1000 person-years.^[11]^ Although no clear data regarding individuals with diabetic LEAD in China are available, local epidemiological investigations and hospital surveys indicated incidence rates for diabetic LEAD of 5.2% to 23.8% among individuals above 50 years of age.^[12–14]^

Timely detection and treatment of LEAD are essential in preventing amputation, morbidity, and mortality of diabetic patients. Although LEAD represents an independent predicting factor of cardiovascular and cerebrovascular ischemia, its diagnosis and treatment remain suboptimal.^[15]^ Hence, diabetic patients need to identify lower-limb atherosclerosis at its early stage.

The association of LEAD with different risk factors of atherosclerosis in T2DM needs continuous investigation. Several risk factors of LEAD have been identified, including age, female sex, years of diabetes, elevated glycosylated hemoglobin (HbA1c), retinopathy, waist-to-hip ratio (WHR), triglyceride (TG) levels, and hypertension.^[16–19]^ A previous study showed that blood lipid, body mass index, smoking, and type of antidiabetic treatment were not associated with LEAD in diabetic patients.^[19]^ However, conventional risk factors for atherosclerosis such as smoking, hypertension, total cholesterol (TC), high-density lipoprotein cholesterol (HDL-C), and TG could not be ignored as risk factors. Hence, this study aimed to identify the risk factors associated with LEAD in the Chinese diabetic population.

In previous studies, diagnosis of peripheral artery disease mostly relied on the ankle brachial index.^[20]^ However, color Doppler ultrasound has many advantages over pressure measurements in that it can both diagnose and localize arterial lesions accurately. Moreover, color Doppler ultrasound correctly differentiates iliac from femoro-popliteal disease, with an overall diagnostic accuracy of 90% in the femoral and popliteal vessels for both occlusion and stenosis.^[21]^ Hence, in the present study, LEAD was evaluated by color Doppler ultrasonography to identify the risk factors associated with LEAD in Chinese individuals with T2DM.

## 2 Patients and methods

### 2.1 Patients

In this case-control study, inpatients with T2DM (n = 1289) admitted at the Department of Endocrinology, Shanghai Jiao Tong University Affiliated Sixth People's Hospital, between January 2008 and December 2009, were enrolled. The subjects were chosen based on World Health Organization guidelines on diagnosis and classification of diabetes mellitus, 1999.^[22]^ There were 746 males and 543 females averagely aged 61 (22–90) years, with a median T2DM course of 6 (0.01, 40) years. Patients with autoimmune disease, acute infections, acute complication of diabetes mellitus, primary renal disease, active stage of renal stone, and other serious diseases were excluded. In addition, patients receiving treatment with glucocorticoids and immuno-suppressors were also excluded. This study was approved by the Ethics Committee of Shanghai Jiao Tong University Affiliated Sixth People's Hospital. Signed informed consent was provided by each participant, and all principles of the Declaration of Helsinki were applied.

### 2.2 Determination of variables

Clinical and demographic data of patients, including age, sex, diabetes duration, body mass index, WHR, microalbuminuria (MAU), glomerular filtration rate (GFR), creatinine, and history of hypertension, dyslipidemia, coronary heart disease (CHD), and cerebral vascular disease (CVD) were collected. Patients’ medical data at admission included waist circumference, hip circumference, and blood pressure assessments. WHR was calculated as waist circumference/hip circumference. Hypertension was reflected by diastolic blood pressure ≥90 mm Hg, systolic blood pressure ≥140 mm Hg, or current use of antihypertensive medication. Dyslipidemia was determined by any of the following criteria: elevated TC ≥6.22 mmol/L and/or TG ≥2.27 mmol/L, elevated low-density lipoprotein cholesterol (LDL-C) ≥4.14 mmol/L, and decreased HDL-C <1.04 mmol/L (according to the American National Cholesterol Education Program, Adult Treatment Panel III),^[23]^ or patients under lipid-lowering drugs.^[24]^ CHD was considered with a history of ischemia or abnormal electrocardiogram findings. CVD was considered with a history of ischemia or stroke. The diagnosis of diabetic peripheral neuropathy (DPN) was according to the Standards of Medical Care in Diabetes, 2008, published by the American Diabetes Association.^[25]^ The diagnosis of diabetic retinopathy (DR) was based on the International Clinical Diabetic Retinopathy and Diabetic Macular Edema Disease Severity Scales.^[26]^ GFR was assessed by radionuclide renal dynamic imaging (modified Gate method) after intravenous injection of ^99^mTc-diethylene-triamine-penta-acetic acid and using a Siemens Signature e.cam single photon emission computed tomography (Siemens, Erlangen, Germany).

### 2.3 Laboratory tests

Fasting blood specimens were collected before breakfast the day after admission for fasting plasma glucose (FPG), HbA1c, uric acid (UA), TC, TG, LDL-C, and HDL-C assessment. Plasma glucose levels were determined by the glucose oxidase method (Automatic Biochemistry Analyzer; Beckman Coulter). Serum lipid components and renal function parameters were assessed by routine procedures on an AutoAnalyzer (Hitachi 7600–020; Hitachi, Tokyo, Japan). HbA1c was estimated by high-performance liquid chromatography on a HLC-723G7 analyzer (Tosoh Corporation, Japan).

### 2.4 Determination of LEAD

Color duplex ultrasonography was performed by 3 experienced sonographers using an ACUSON Sequoia 512 ultrasound system (Siemens, Erlangen, Germany), with a 5 to 13-MHz linear transducer, based on a previously published technique.^[27]^ Femoral intima-media thickness (FIMT) values for both sides were determined as the distance from the leading edge of the lumen-intima echo to that of the media-adventitia echo. LEAD was considered with the observation of a focal structure intruding in the arterial lumen by 0.5 mm or ≥50% thicker than the surrounding vessel wall, or intima-media thickness (IMT) >1.5 mm in any lower-limb arteries according to the Mannheim IMT consensus.^[28]^ In subgroup analysis, patients with T2DM and LEAD were categorized into 2 groups: LEAD+A (1%–49% diameter of lower-limb arteries reduction) and LEAD+B (≥50% diameter reduction).

### 2.5 Statistical analysis

The statistical analysis was performed using SPSS 18.0 (IBM, Armonk, NY). Normally distributed quantitative variables were presented as mean ± standard deviation (SD) and compared using independent-samples *t* test; data with non-normal distribution were presented as median (range) and evaluated using Mann–Whitney *U* test. Categorical data were expressed as frequency and compared using the chi-square test. Multivariate logistic regression analysis was performed to determine the odds ratios (OR) and 95% confidence intervals (95% CIs) using the enter method. Independent variables with *P* < 0.05 in univariate analyses were entered in the multivariate logistic regression. *P* < 0.05 was considered statistically significant.

## 3 Results

### 3.1 Comparison of clinical characteristics among different LEAD groups

Compared with the LEAD− group, patient age, course of diabetes mellitus, WHR, serum creatinine, HDL-C level, MAU, hypertension, systolic blood pressure, CHD, and CVD in the LEAD+ group were significantly higher, whereas the levels of FPG, GFR, and TG were significantly lower (all *P* < 0.05). Sex, BMI, level of HbA1C, UA, LDL-C, TC, diastolic pressure, rate of dyslipidemia, DR, and DPN were not significantly different (all *P* > 0.05) (Table 1). In subgroup analysis, compared with the LEAD+A group, patient age, course of diabetes mellitus, WHR, UA levels, serum creatinine, and systolic blood pressure in the LEAD+B group were significantly higher, whereas GFR, hypertension rate, DR, CHD, and CVD were markedly lower (all *P* < 0.05). Sex, BMI, level of FPG, HbA1C, MAU, HDL-C, LDL-C, TC, TG, diastolic pressure, dyslipidemia, and DPN were not significantly different (all *P* > 0.05) (Table 1).

**Table 1:**
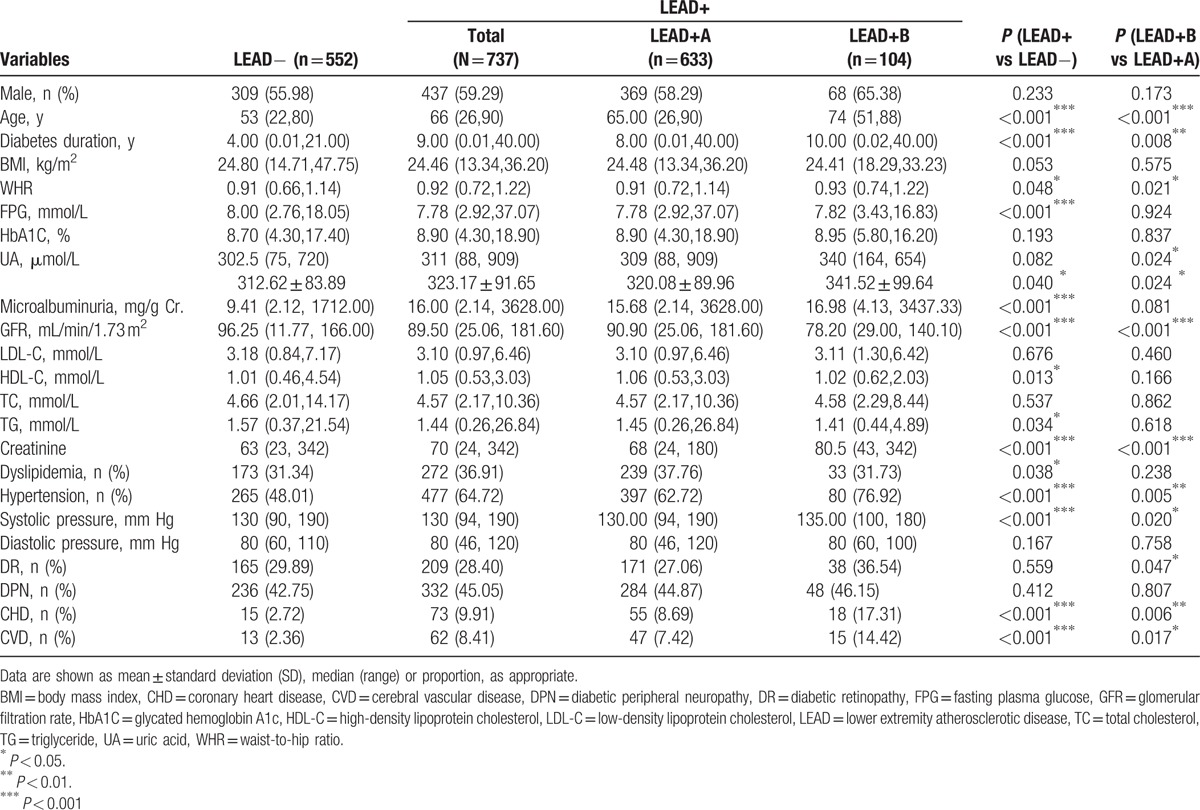
Comparison of clinical parameters among LEAD groups.

### 3.2 Multivariate logistic regression analysis of independent factors associated with LEAD in patients with T2DM

Independent variables including age, diabetes duration, WHR, FPG, creatinine, UA, MAU, GFR, dyslipidemia, and hypertension were evaluated using a multivariate logistic regression model. GFR (OR 0.991, 95% CI 0.986–0.997, *P* = 0.003), diabetes duration (OR 1.055, 95% CI 1.026–1.084, *P* < 0.001), age (OR 1.123, 95% CI 1.104–1.142, *P* < 0.001), and UA (OR 1.002, 95% CI 1.000–1.004, *P* = 0.031) were independently associated with LEAD in patients with T2DM (Table 2).

**Table 2:**
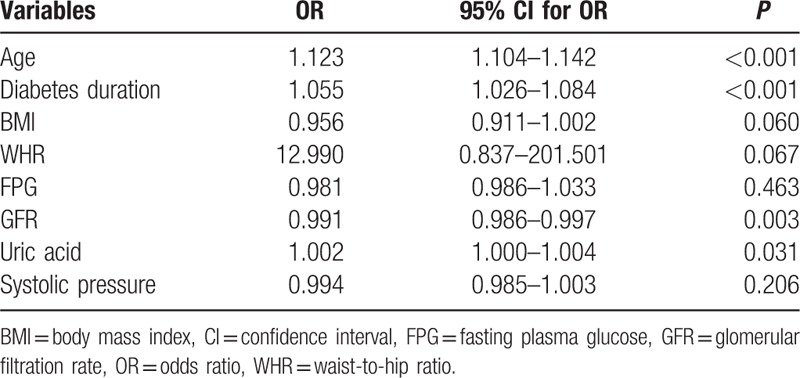
Multivariate logistic regression analysis of independent factors associated with lower extremity atherosclerotic disease in Chinese patients with type 2 diabetes mellitus.

### 3.3 Multivariate logistic regression analysis of independent factors associated with lesion severity in patients with T2DM and LEAD

Independent variables including age, diabetes duration, WHR, creatinine, UA, GFR, hypertension, and DR were evaluated using a multivariate logistic regression model. As shown in Table 3, age (OR 1.078, 95% CI 1.048–1.109, *P* < 0.001) and GFR (OR 0.985, 95% CI 0.975–0.994, *P* = 0.002) were independently associated with the severity of arterial lesions in patients with T2DM and LEAD (Table 3).

**Table 3:**
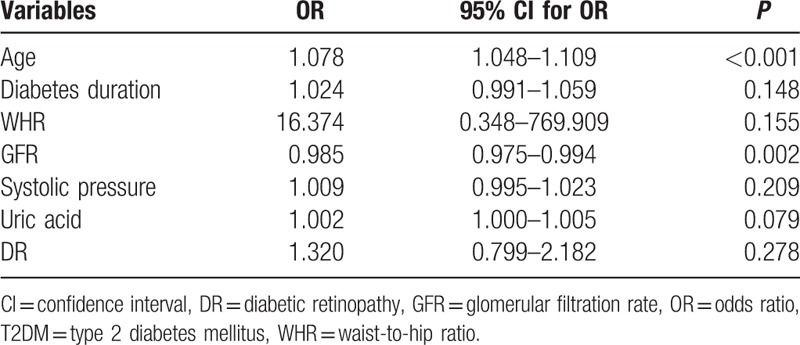
Multivariate logistic regression analysis of independent factors associated with the severity of the lesion in patients with T2DM and lower extremity atherosclerotic disease.

## 4 Discussion

In the present study, age, GFR, duration of diabetes, and UA were found to be independent risk factors of LEAD in patients with T2DM. In addition, age and GFR were independently associated with lesion severity in patients with T2DM and LEAD.

The prevalence of LEAD is overtly higher in the diabetic population than in nondiabetic individuals, and LEAD substantially increases the risk of cardiovascular and cerebrovascular events such as myocardial infarction and stroke.^[29,30]^ Furthermore, cardiovascular and cerebrovascular event rates are greater in diabetic individuals, with atherosclerosis compared with comparable nondiabetic populations.^[31]^ Diabetic patients with peripheral arterial disease comorbidity are at higher risk of lower-extremity amputation than those without diabetes.^[32]^ Hence, timely detection and treatment of LEAD is essential in preventing amputation and death in diabetic patients.

The development of LEAD in T2DM involves many aspects, among which patient age and course of diabetes are risk factors.^[33–35]^ In agreement, the present study also showed that the risk factors for LEAD in T2DM included age and diabetes duration. The possible reasons could be as follows: the incidence of arteriosclerosis increases with age; and prolonged disease course indicates chronic hyperglycemia, and extended chronic hyperglycemia contributes to protein glycation and yields advanced glycation end products, and also oxygen free radicals, which damage vascular endothelial cells and promote arteriosclerosis, and also plaque formation.

In the present study, FPG was lower in patients with LEAD, but not identified as an independent predicting factor of LEAD, in disagreement with previous reports.^[34,35]^ The possible reasons could be that the vast majority of patients assessed here were hospitalized for severely poor glycemic control, and FPG levels at admission might differ from the values obtained in the general population.

Hyperuricemia is a well-known risk factor for cardiovascular diseases.^[36,37]^ Indeed, Chuengsamarn et al^[38]^ found that elevated UA levels are significantly associated with diabetes-related chronic micro/macrovascular complications. Therefore, UA levels monitoring has a prognostic value for chronic micro/macrovascular complications in diabetic patients. Zhang et al^[39]^ investigated 2174 T2DM individuals and found serum UA levels to be an independent risk factor for LEAD in female diabetic patients. These reports corroborated our findings that serum UA levels were higher in diabetic patients with LEAD, and represented a significant predicting factor for LEAD. In the present study, hypertension rate, CHD, and cerebral infarction rate were markedly higher in diabetic patients with LEAD than in those without LEAD, in accordance with previously published data.^[3,34,40]^

There is a certain correlation between macroangiopathy and microangiopathy. Yu et al^[41]^ found that LEAD is related to diabetic nephropathy in T2DM patients. Yap et al^[42]^ demonstrated that GFR decrease is an independent risk factor for diabetic LEAD regardless of proteinuria level, with LEAD incidence in the abnormal GFR group obviously increased compared with that of the normal GFR group. Furthermore, higher incidence of LEAD was evidenced with a more apparent decline of GFR. In the present study, logistic regression analysis showed that UA and GFR were independent risk factors for diabetic LEAD. Dysfunction of endothelial cells in patients with diabetic nephropathy may account for these findings. The dysfunction not only exists in kidney capillaries, but also in systemic vascular beds. Plasma proteins can permeate through damaged endothelial cells and cause changes in the endarterium, further promoting the development of atherosclerosis.

In the present study, subgroup analysis results showed that age and GFR were independent risk factors for lesion severity in T2DM patients with LEAD. In a previous study, age was found to be an independent risk factor for the severity of peripheral arterial disease.^[43]^

A number of factors are involved in the development of LEAD and were not evaluated in the present study. Indeed, recent reports demonstrated the role of infectious diseases in peripheral artery disease. Indeed, it was suggested that periodontal pathogens might translocate from subgingival microbiota to the bloodstream, further progressing to atheromatous plaques in peripheral arteries.^[44]^ In addition, *Salmonella* infects peripheral or visceral arteries, and most commonly the abdominal aorta,^[45]^ and multiple sexually transmitted diseases such as Syphilis and acquired immunodeficiency syndrome (AIDS) were shown to be involved in vascular diseases.^[46,47]^ However, this factor was not evaluated in the present study because of difficulties in identifying all infections, especially those without overt clinical signs, and to distinguish acute from chronic infections. Additional studies are necessary to assess this issue.

A few limitations of the present study should be mentioned. First, we could not analyze the progression of atherosclerotic lesions. In addition, the study was carried out in a single center, with a small sample size. Finally, other confounding factors of LEAD were not excluded. Therefore, additional larger clinical studies are warranted to confirm the risk factors of LEAD reported in this study.

## 5 Conclusions

Lower extremity atherosclerotic disease in T2DM patients is a chronic pathological process caused by multiple factors. Risk factors for LEAD include age, disease course, UA levels, and GFR. Patients with T2DM, high UA levels, and declined GFR should be considered a high-risk group for LEAD.

## References

[1] Yang W Lu J Weng J Prevalence of diabetes among men and women in China. N Engl J Med 2010;362:1090–101. 20335585 10.1056/NEJMoa0908292

[2] Scholte AJ Schuijf JD Kharagjitsingh AV Prevalence of coronary artery disease and plaque morphology assessed by multi-slice computed tomography coronary angiography and calcium scoring in asymptomatic patients with type 2 diabetes. Heart 2008;94:290–5. 17646190 10.1136/hrt.2007.121921

[3] Balogh O Pentek M Gulacsi L [Quality of life and burden of disease in peripheral arterial disease: a study among Hungarian patients]. Orv Hetil 2013;154:464–70. 23506803 10.1556/OH.2013.29567

[4] Dhaliwal G Mukherjee D Peripheral arterial disease: epidemiology, natural history, diagnosis and treatment. Int J Angiol 2007;16:36–44. 22477268 10.1055/s-0031-1278244 PMC2733014

[5] Kullo IJ Bailey KR Kardia SL Ethnic differences in peripheral arterial disease in the NHLBI Genetic Epidemiology Network of Arteriopathy (GENOA) study. Vasc Med 2003;8:237–42. 15125483 10.1191/1358863x03vm511oa

[6] Vogt MT Cauley JA Kuller LH Functional status and mobility among elderly women with lower extremity arterial disease: the Study of Osteoporotic Fractures. J Am Geriatr Soc 1994;42:923–9. 8064098 10.1111/j.1532-5415.1994.tb06581.x

[7] McDermott MM Liu K Greenland P Functional decline in peripheral arterial disease: associations with the ankle brachial index and leg symptoms. JAMA 2004;292:453–61. 15280343 10.1001/jama.292.4.453

[8] Adler AI Boyko EJ Ahroni JH Lower-extremity amputation in diabetes. The independent effects of peripheral vascular disease, sensory neuropathy, and foot ulcers. Diabetes Care 1999;22:1029–35. 10388962 10.2337/diacare.22.7.1029

[9] Dickinson PJ Carrington AL Frost GS Neurovascular disease, antioxidants and glycation in diabetes. Diabetes Metab Res Rev 2002;18:260–72. 12203942 10.1002/dmrr.305

[10] Umuerri EM Obasohan AO Lower extremity peripheral artery disease: prevalence and risk factors among adult Nigerians with diabetes mellitus. West Afr J Med 2013;32:200–5. 24122686

[11] Li L Yu H Zhu J The combination of carotid and lower extremity ultrasonography increases the detection of atherosclerosis in type 2 diabetes patients. J Diabetes Complications 2012;26:23–8. 22226486 10.1016/j.jdiacomp.2011.11.006

[12] Touboul PJ Hennerici MG Meairs S Mannheim intima-media thickness consensus. Cerebrovasc Dis 2004;18:346–9. 15523176 10.1159/000081812

[13] Lacle A Valero-Juan LF Diabetes-related lower-extremity amputation incidence and risk factors: a prospective seven-year study in Costa Rica. Rev Panam Salud Publica 2012;32:192–8. 23183559 10.1590/s1020-49892012000900004

[14] Li X Wang YZ Yang XP Prevalence of and risk factors for abnormal ankle-brachial index in patients with type 2 diabetes. J Diabetes 2012;4:140–6. 22078109 10.1111/j.1753-0407.2011.00171.x

[15] Marso SP Hiatt WR Peripheral arterial disease in patients with diabetes. J Am Coll Cardiol 2006;47:921–9. 16516072 10.1016/j.jacc.2005.09.065

[16] Selvin E Erlinger TP Prevalence of and risk factors for peripheral arterial disease in the United States: results from the National Health and Nutrition Examination Survey, 1999–2000. Circulation 2004;110:738–43. 15262830 10.1161/01.CIR.0000137913.26087.F0

[17] Guerchet M Aboyans V M’Belesso P 241 Particularities of the epidemiology of lower-extremities peripheral artery disease in Central Africa. Arch Cardiovasc Dis Suppl 2010;2:77–177.

[18] Okello S Millard A Owori R Prevalence of lower extremity peripheral artery disease among adult diabetes patients in southwestern Uganda. BMC Cardiovasc Disord 2014;14:75. 24913468 10.1186/1471-2261-14-75 PMC4057935

[19] Katsilambros NL Tsapogas PC Arvanitis MP Risk factors for lower extremity arterial disease in non-insulin-dependent diabetic persons. Diabet Med 1996;13:243–6. 8689845 10.1002/(SICI)1096-9136(199603)13:3<243::AID-DIA69>3.0.CO;2-U

[20] Rac-Albu M Iliuta L Guberna SM The role of ankle-brachial index for predicting peripheral arterial disease. Maedica (Buchar) 2014;9:295–302. 25705296 PMC4306002

[21] Baxter GM Polak JF Lower limb colour flow imaging: a comparison with ankle: brachial measurements and angiography. Clin Radiol 1993;47:91–5. 8435971 10.1016/s0009-9260(05)81179-1

[22] World Health Organization Definition, diagnosis, and classification of diabetes mellitus and its complications: Report of a WHO consultation. Part 1. Diagnosis and Classification of Diabetes Mellitus. Geneva:World Health Organization; 1999.

[23] Expert Panel on Detection E Treatment of high blood cholesterol in A. Executive summary of the Third Report of The National Cholesterol Education Program (NCEP) Expert Panel on Detection, Evaluation, And Treatment of High Blood Cholesterol In Adults (Adult Treatment Panel III). JAMA 2001;285:2486–97. 11368702 10.1001/jama.285.19.2486

[24] Yan L Xu MT Yuan L Prevalence of dyslipidemia and its control in type 2 diabetes: a multicenter study in endocrinology clinics of China. J Clin Lipidol 2016;10:150–60. 26892132 10.1016/j.jacl.2015.10.009

[25] American Diabetes Association Standards of medical care in diabetes: 2008. Diabetes Care 2008;31 (suppl 1):S12–54. 18165335 10.2337/dc08-S012

[26] Zhang J Xue YM [The risk factors for abnormal ankle-brachial index in type 2 diabetic patients and clinical predictive value for diabetic foot]. Zhonghua Nei Ke Za Zhi 2013;52:951–5. 24439190

[27] Guan H Li YJ Xu ZR Prevalence and risk factors of peripheral arterial disease in diabetic patients over 50 years old in China. Chin Med Sci J 2007;22:83–8. 17763578

[28] Montero-Monterroso JL Gascon-Jimenez JA Vargas-Rubio MD [Prevalence and factors associated with peripheral artery disease in patients with type 2 diabetes mellitus in Primary Care]. Semergen 2015;41:183–90. 25042974 10.1016/j.semerg.2014.05.004

[29] Weitz JI Byrne J Clagett GP Diagnosis and treatment of chronic arterial insufficiency of the lower extremities: a critical review. Circulation 1996;94:3026–49. 8941154 10.1161/01.cir.94.11.3026

[30] Dormandy JA Rutherford RB Management of peripheral arterial disease (PAD). TASC Working Group. TransAtlantic Inter-Society Consensus (TASC). J Vasc Surg 2000;31 (1 Pt 2):S1–296. 10666287

[31] American Diabetes Association Peripheral arterial disease in people with diabetes. Diabetes Care 2003;26:3333–41. 14633825 10.2337/diacare.26.12.3333

[32] Jude EB Oyibo SO Chalmers N Peripheral arterial disease in diabetic and nondiabetic patients: a comparison of severity and outcome. Diabetes Care 2001;24:1433–7. 11473082 10.2337/diacare.24.8.1433

[33] Wongkongkam K Thosingha O Riegel B Factors influencing the presence of peripheral arterial disease among Thai patients with type 2 diabetes. Eur J Cardiovasc Nurs 2012;11:70–6. 22357781 10.1177/1474515111429658

[34] Althouse AD Abbott JD Forker AD Risk factors for incident peripheral arterial disease in type 2 diabetes: results from the Bypass Angioplasty Revascularization Investigation in type 2 Diabetes (BARI 2D) Trial. Diabetes Care 2014;37:1346–52. 24595631 10.2337/dc13-2303 PMC3994929

[35] Xu J Wang G Fu D High-resolution color Doppler ultrasound examination and related risk factor analysis of lower extremity vasculopathy in type 2 diabetes patients. Genet Mol Res 2015;14:3939–47. 25966165 10.4238/2015.April.27.8

[36] Yoo JH Park JE Hong KP Moderate hyperhomocyst(e)inemia is associated with the presence of coronary artery disease and the severity of coronary atherosclerosis in Koreans. Thromb Res 1999;94:45–52. 10213180 10.1016/s0049-3848(98)00197-2

[37] Kanbay M Yilmaz MI Sonmez A Serum uric acid independently predicts cardiovascular events in advanced nephropathy. Am J Nephrol 2012;36:324–31. 23007099 10.1159/000342390

[38] Chuengsamarn S Rattanamongkolgul S Jirawatnotai S Association between serum uric acid level and microalbuminuria to chronic vascular complications in Thai patients with type 2 diabetes. J Diabetes Complications 2014;28:124–9. 24412514 10.1016/j.jdiacomp.2013.12.002

[39] Zhang L Zhou J Li Q [Association between serum uric acid level and peripheral vascular disease of lower extremities in type 2 diabetes mellitus subjects]. Zhonghua Yi Xue Za Zhi 2010;90:653–7. 20450721

[40] Lai YJ Hu HY Lin CH Incidence and risk factors of lower extremity amputations in people with type 2 diabetes in Taiwan, 2001–2010. J Diabetes 2015;7:260–7. 24823436 10.1111/1753-0407.12168

[41] Yu JH Hwang JY Shin MS The prevalence of peripheral arterial disease in Korean patients with type 2 diabetes mellitus attending a university hospital. Diabetes Metab J 2011;35:543–50. 22111047 10.4093/dmj.2011.35.5.543 PMC3221031

[42] Yap YS Chuang HY Chien CM Relationship between peripheral artery disease and combined albuminuria and low estimated glomerular filtration rate among elderly patients with type 2 diabetes mellitus. Diab Vasc Dis Res 2014;11:41–7. 24227538 10.1177/1479164113510924

[43] Newman AB Sutton-Tyrrell K Rutan GH Lower extremity arterial disease in elderly subjects with systolic hypertension. J Clin Epidemiol 1991;44:15–20. 1986053 10.1016/0895-4356(91)90196-g

[44] Figuero E Lindahl C Marin MJ Quantification of periodontal pathogens in vascular, blood, and subgingival samples from patients with peripheral arterial disease or abdominal aortic aneurysms. J Periodontol 2014;85:1182–93. 24502612 10.1902/jop.2014.130604

[45] Cohen JI Bartlett JA Corey GR Extra-intestinal manifestations of salmonella infections. Medicine (Baltimore) 1987;66:349–88. 3306260 10.1097/00005792-198709000-00003

[46] Pavithran K Syphilitic peripheral vascular disease: a case report. Indian J Sex Transm Dis 1989;10:79–81. 12284237

[47] Chetty R Batitang S Nair R Large artery vasculopathy in HIV-positive patients: another vasculitic enigma. Hum Pathol 2000;31:374–9. 10746682 10.1016/s0046-8177(00)80253-1

